# Social Quality and Health: Examining Individual and Neighbourhood Contextual Effects Using a Multilevel Modelling Approach

**DOI:** 10.1007/s11205-017-1640-2

**Published:** 2017-05-12

**Authors:** Daniel Holman, Alan Walker

**Affiliations:** Department of Sociological Studies, University of Sheffield, Sheffield, S10 2TU UK

**Keywords:** Social quality, Social determinants, Neighbourhood effects, Self-rated health, Multilevel modelling

## Abstract

Social quality focusses on the nature of ‘the social’, arguing that people are realised as social beings through interacting with a range of collectives, both from the formal world of systems and the informal lifeworld. Four conditional factors are necessary for this to occur, which at the same time are assumed to influence health and well-being: socio-economic security, social cohesion, social inclusion and social empowerment. In this paper we test the utility of social quality in explaining self-rated health as a response to arguments that the social determinants of health (SDH) framework often lacks a theoretical basis. We use multilevel models to analyse national English and Welsh data (the Citizenship Survey) to test for both individual- and neighbour-level affects. Our key findings are that (1) neighbourhood contextual (cross-level) effects are present with respect to collective action, personal trust, cross-cutting ties, income sufficiency, and income security; (2) measures of national, community and personal identity as indicators of social cohesion show clear associations with health alongside more common measures such as trust; (3) the security aspects of socioeconomic determinants are especially important (housing security, income sufficiency, and income security); (4) social rights, including institutional rights but especially civil rights have effects of particularly large magnitude. Social quality offers a theoretically-driven perspective on the SDH which has important policy implications and suggests a number of promising avenues for future research.

## Introduction

Following the seminal UK Black Report (1980) evidence has been building on the social factors underpinning health. In recent years the social determinants of health (SDH) framework has been central to this research (Marmot and Wilkinson 2005), and the World Health Organisation’s highly influential report was a landmark (CSDH 2008). The SDH framework is an empirically-led endeavour concerned with identifying risk factors from a social epidemiological perspective. In this field, theoretical explanations have taken three main directions: psychosocial approaches, social production of disease/political economy of health, and eco-social frameworks (Solar and Irwin 2010:15). *Psychosocial* approaches focus on the idea that the ‘perception and experience of personal status in unequal societies lead to stress and poor health’ (*ibid*.). *Social production of disease/political economy of health* focusses on wider economic and political determinants, especially the structural causes of inequalities i.e. the unequal distribution of resources. *Eco*-*social* explanations conceive of health as complex, multi-layered, and dynamic, and mutually constituted by the biological, psychological, and wider organisation of society.

Although these explanations have led to important insights, it has been argued that social epidemiology would benefit from greater use of theory. For example, in an important paper on the discipline, Galea and Link (2013:847) argue that the field needs:

deeper engagement for the field in theory, a richer grounding in an understanding of why particular factors may matter, and the confidence to articulate a priori hypotheses about what social conditions might matter, leading to testing through observational or experimental studies.

Some have argued that there has been too much focus on psychosocial approaches (Coburn 2004; Peacock et al. 2014), especially the income inequality hypothesis [the idea that income inequality leads to poor health through lower levels of cohesion, trust, sense of control and shame/pride (Wilkinson 2006)], and sociological insights on class and welfare are much needed (Graham 2007). This is not to detract from the increasing attention to wider political determinants such as the current neo-liberal political climate (Schrecker and Bambra 2015) and recent financial crisis (e.g. Reeves et al. 2014).

Social quality offers a new framework for exploring SDH, answering the need for theoretical enrichment, especially from outwith social epidemiology. Instead of being empirically-driven and focussing on risk factors, it is theoretically-led, taking the nature of the social itself as its starting point. Broadly, it argues that the realisation of social life, entailing participation and recognition, is fundamentally important for health and well-being. After briefly outlining social quality theory, we then compare it with the SDH framework, review existing research on area-level explanations for health, and summarise previous empirical findings on social quality.

### Social Quality

The concept of social quality emerged in the 1990s in response to scientific and political concerns about the dominance of economism in debates about the future of the European Union (EU) as well as in those taking place within several member states, including the UK (Beck et al. 1998). It was clear then and, arguably, is even clearer now, that the imperatives of neo-liberalism were driving out any serious consideration of the social dimensions of both EU and national policy making. In a nutshell the idea behind social quality was to bring the social back in (van der Maesen and Walker 2012). This analysis and conceptualisation of social quality owed much to earlier critiques of economic imperialism (Walker 1984), the subordination of social policy to economic policy (Titmuss 1974) and critical philosophical investigations into the nature of the social (Bhaskar 1978; Elias 2000; Habermas 1989). All of them, of course, were oppositional to the assertion that there is no such thing as the social (Hayek 1988).

Thus the starting point for social quality is the essentially social nature of human life, in contrast to the atomised individualism of neo-liberalism. The realm of the social consists of the twin endeavours of self-realisation and the creation of the myriad collectivities within which it is achieved. In other words individual identity is shaped by society through the process of social recognition (Honneth 1995). Behind the interplay between self-identity and collective identities are two sets of tensions: between individual or biographical development and societal development (micro vs macro) and between institutions and organisations, on the one hand, and families, groups and communities on the other (system and lifeworld). For this social process to take place in any locality or society there have to be some basic requisites: social recognition or mutual respect; human rights and the rule of law (personal security); individual competence (the ability to act socially); and the openness of social groups/collectivities (social responsiveness) are the obvious ones. The definition of social quality reflects these various assumptions: ‘the extent to which people are able to participate in the social, economic and cultural lives of their communities under conditions which enhance their well-being and individual potential’ (Beck et al. 1998:4).

As well as a theoretical foundation, summarised drastically here, social quality has a distinct empirical orientation. This emphasises four empirical conditional factors which govern the extent and quality of social participation:

- *Socio*-*economic security* command over material and other resources over time.
- *Social cohesion* the extent to which norms and values are accepted and shared.
- *Social inclusion* the extent to which people have access to and are integrated into a wide variety of institutions and social relations.
- *Social empowerment* how far social structures, relations and institutions enable individuals to participate and develop their capabilities.

**Fig. 1:**
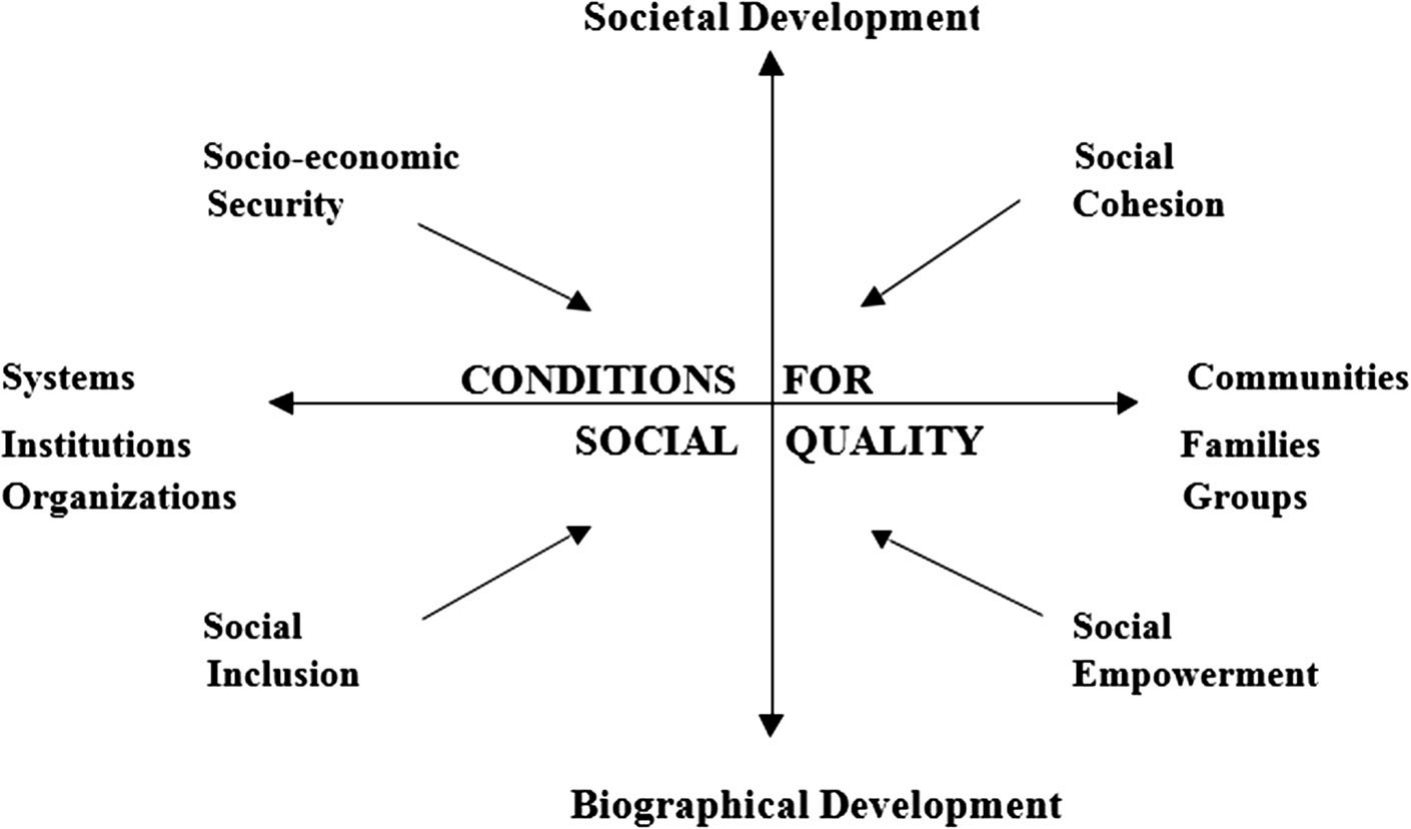
The conditional factors of social quality

These four conditional factors and the framing structure of social quality outlined above are illustrated in Fig. 1.

This model has been operationalised successfully in cross-national comparative research in Europe (East and West) (Abbott et al. 2010; Abbott and Wallace 2012, 2014) and East Asia (Lin 2014; Yee and Chang 2011) but is only now being applied to solely British data sets. This application is timely because social quality is the only comprehensive model designed to evaluate the quality of society, as opposed to the wide variety of measures of quality of life at the individual level (Phillips 2006). The much-employed concept of social capital focusses primarily on relational stocks accrued by individuals rather than the role of society (van der Maesen and Walker 2012:253). Moreover social quality is particularly well suited for the investigation of health outcomes. Its definition specifically refers to well-being and, as we go on to explore, each of its conditional factors has implications for health.

#### The Relationship Between the Social Quality and Social Determinants of Health Frameworks

Whereas the SDH framework concentrates on risk factors for health, social quality is in essence concerned with risk factors for social participation and realisation, which in turn are assumed to influence health and well-being. For example, in the WHO report on SDH social empowerment is often discussed in terms of health empowerment (2008:96). Whilst it has also been argued that political empowerment is a central social determinant of health (Marmot et al. 2008), it has received much less attention than other concepts in the framework. Under social quality empowerment is a fundamental conditional factor, encompassing political empowerment but also how socially empowered people are across all areas of society. Similarly, the focus on income inequality, and specifically how it is thought to erode social cohesion, is a specific concern of SDH. Under social quality, income inequality conceivably erodes not only social cohesion, but the other three conditional factors; it lessens socioeconomic security, closes off institutions/relations, and hinders the extent to which institutions/relations empower individuals.

To be sure, despite their different focus, there are obvious theoretical overlaps between the SDH and social quality frameworks. Social quality aligns most with the second theoretical direction of SDH—the social production of disease, or what has been termed the neo-materialist position. In short this position suggests that:

Economic processes and political decisions condition the private resources available to individuals and shape the nature of public infrastructure – education, health services, transportation, environmental controls, availability of food, quality of housing, occupational health regulations – that forms the “neomaterial” matrix of contemporary life. Thus income inequality per se is but one manifestation of a cluster of material conditions that affect population health (CSDH 2008:16).

What sets social quality apart from this position is the focus on *social* resources. The underlying causal mechanism centres around the (four) conditional factors which are assumed to provide the social conditions necessary for people to realise themselves as social beings—a fundamental aspect of well-being given the social nature of human life.

The frameworks also share some similarities in their conceptualisations: as with SDH, indicators of social position such as education, gender, and ethnicity are seen as affecting access to resources. Social cohesion features in both frameworks, but under social quality it is one of four conditional factors rather than being an intermediary determinant alongside material circumstances, psychosocial factors, behaviours and biological factors (CSDH 2008).

Empirically, given the shared concerns between social quality and SDH, both frameworks, to some extent, share common indicators, which have been examined in previous research. For example, much attention has been paid to the health effects of trust (Subramanian et al. 2002), social networks (Poortinga 2012), and socioeconomic factors. At the same time, missing is a common conceptual framework to link these factors together (Ward et al. 2011). Furthermore, little attention has been paid to social empowerment, identity and rights as social determinants. In this paper we include the range of indicators suggested by social quality for completeness and comparison, but in interpreting the findings concentrate on the more novel indicators.

#### Area-Level Effects on Health

A second area of research highly relevant to the social quality agenda is that on the area-level effects on health. Social quality naturally lends itself to area-level research since it focuses on underlying conditions which are thought to benefit the social collectivity. Pickett and Pearl highlighted in 2001 how the topic had seen increased attention as a result of an interest in societal influences on health combined with improved statistical techniques (2001:111). The field is difficult to summarise due to its heterogeneity, namely in terms of conceptual and methodological issues, as comprehensively outlined by Riva et al. (2007). This evaluation leads the authors to conclude that a ‘specific’ research approach is needed:

the adoption of a specific research approach to examine area effects on health – that is, one that would conceptualise, operationalise, and measure associations between specific health outcomes and specific area exposures – across specific spatial area units may yield more informative evidence of area effects. Adopting a specific approach shows the greatest promise for advancing theoretically based pathways, providing a basis for more precise definitions and measures of ecological exposures, and improved delimitations of area contours (2007:859).

We follow Riva et al.’s suggestion by examining effects using a proxy for neighbourhood, specifying self-rated health as the outcome, and analysing indicators on a case-by-case basis. Given that different factors have different effects depending on the level at which they operate [for example social support might be more important at the neighbourhood level whilst level of healthcare is more important in terms of catchment areas (Pickett and Pearl 2001:112)], we interpret effects specifically with reference to the neighbourhood.

Due to issues of data availability, we aggregate individual measures to examine neighbourhood-level effects, though we are mindful that true area-level effects are also likely to be an important influence. For example, Macintyre et al. (2002), outline five features of local areas which might influence health: physical features including quality of water and climate; availability of healthy environments, including decent housing, safe play areas for children; services including education, transport, street lighting and policing, socio-cultural features including ethnic and religious history of a community, norms, values, integration, and the reputation of an area, including perceptions by residents, amenity planners and investors. There is some overlap with social quality here since it suggests that neighbourhoods matter for health because they provide the local social conditions i.e. social empowerment, cohesion, inclusion, and socio-economic security, that enable people to realise themselves as social beings and experience well-being, though of course in reality social resources influence health at different levels in complex interconnected ways.

#### Existing Research on Social Quality and Health

There are a small number of existing studies examining the relationship between social quality and well-being, though as far as we can tell none specifically on health outcomes, and none considering area-level effects. Some researchers have taken the approach of treating social quality as an outcome showing how its experience is moderated by social position (Ward et al. 2011). Research using social quality as a predictor has tended to take the approach of constructing scales for each of the four conditional factors, but given our focus on area-level effects and our use of secondary data, which limits data availability, we follow the specific research agenda as discussed above. For example, Abbott et al. (2010) constructed scales of each of the four social quality conditional factors using factor analysis from a wide range of relevant variables. They found that economic factors were the strongest predictors of life satisfaction (25% variance explained), followed by social cohesion (20%), social integration (10%) and social empowerment (13%). In an analysis of the EU27 countries, the same authors carried out a series of regression models with a range of variables they selected to measure the social quality concept. Scales were not constructed, rather, the variables were entered in blocks in regression models corresponding with the four conditional factors. The authors found that economic factors explained most variance in life satisfaction, followed by conditions for empowerment, but that cohesion and inclusion also made a contribution (Abbott and Wallace 2012). In a Chinese context, Yuan and Golpelwar (2012) used recommendations from Abbott and Wallace for indicator variables, and analysing these variables separately, found that all four conditional factors of social quality had strong (but differing) links with subjective well-being. In an analysis of survey data from three Chinese cities, Lin (2014) also analysed social quality variables separately, but found that social inclusion was less influential than the other domains, consistent with Abbott et al.’s (2010) study.

In this paper we advance the quality of society debate by clearly specifying the social quality indicators and examining both individual and area-level (neighbourhood) effects. We build upon the argument that social quality is a potentially productive avenue for a sociologically-oriented analysis of the SDH (Ward et al. 2011).

## Methods

### Sample

We analysed data from the Citizenship Survey, which includes a range of variables that map onto the indicators suggested by the social quality framework. The Citizenship Survey was a large, nationally representative cross-sectional survey covering England and Wales that ran every 2 years from 2001 until 2011. Data from the 2011 survey were analysed with a sample size of 8139 individuals.

### Health Outcomes

Self-rated health has been shown to be a good predictor of mortality for people with a range of socio-economic backgrounds and health circumstances (Burström and Fredlund 2001), and was recently found to be the best predictor of mortality for men aged 37–73 years amongst 655 measurements in an analysis of the UK Biobank (Ganna and Ingelsson 2015). Self-rated health was measured on a five-point scale from very good to very bad. Although sometimes dichotomised and modelled as a binary variable, dichotomisation leads to loss of information. We therefore used ordered logit regression models to analyse self-rated health on the original scale (Manor et al. 2000) (see below).

### Socio-demographic and Socioeconomic Variables

The models including both individual and neighbourhood aggregated social quality were adjusted in two steps: first for individual socio-demographic variables [gender, age, ethnicity (BME indicator), income (eight categories), education (highest level of qualification—four levels), and the four category version of the National Statistics Socio-economic Classification (NS-SEC), a measure of occupational social class], and second for neighbourhood-level factors [the Index of Multiple Deprivation (IMD)—a composite indicator comprising seven aspects of deprivation, and a neighbourhood urban/rural indicator]. We control for these factors because previous research suggests they might confound the relationship between social quality and health. IMD was coded into quintiles, and England and Wales scores were combined. Unfortunately, an error with the dataset meant that education data were missing for those aged 70+, restricting the analysis to those aged 16–69. All variables were modelled using dummy categories, except age and income, which were modelled as continuous variables. An age squared term was included to control for non-linear age effects. Missing data for these variables was negligible, except for income where 10% of the sample refused to answer this question or answered ‘Don’t know’, which meant that the fully adjusted models did not include these respondents. However, income was kept in the analysis because it is a potentially important confounder of the links between social quality and health.

### Social Quality Indicators

Overall, the Citizenship Survey had coverage for 5/14 sub-domains for social empowerment, 9/11 for social cohesion, 6/12 for socio-economic security, and 4/16 for social inclusion (see Table 1). The selected variables were measured using a mixture of dichotomous and Likert-type response categories. Multiple indicators were used for each sub-domain of social quality (e.g. institutional trust) where available, and these were summed together if they were all measured using the same scale. Where there was a mixture of response scales, Likert-type questions were dichotomised (details for all variables are available in “Appendix”). This meant that the different sub-domains had different ranges. To enable comparability, we applied Gelman’s (2008) method of scaling non-dichotomous variables by dividing by two standard deviations, which makes non-dichotomous coefficients approximately comparable with dichotomous coefficients. In the case of binary variables, this transformation was applied to the neighbourhood aggregates. Because this is an approximate method, it means that effect sizes cannot be taken as precise. To model neighbourhood effects, individual responses (of the scaled inputs) were aggregated at the neighbourhood level. Finally, we labelled the indicators as to whether they related to respondents’ perceptions, or reported experiences, since each may have unique causal pathways/mechanisms with health. For example, the perception that one has social support can be as important as the amount of support one actually has (Turner 1999). However, we acknowledge that this distinction is not always clear cut.

**Table 1:** Social quality indicators

Domain/sub-domain	Indicators	Number (%)
*Social empowerment*		
Application of knowledge (perceived)	Job fully uses skills/qualifications	2105/5169 (40.72%)
Availability of information (perceived)	Very good at reading English	7206/8129 (88.65%)
	Very good at writing English	7080/8131 (87.07%)
Openness and supportiveness of political system (experienced)	Taken part in a consultation about local services	1493/8137 (18.35%)
	Contacted a political representative	1937/8137 (23.80%)
	Been a member of a group making decisions on services	759/8139 (9.33%)
Openness and supportiveness of political system (perceived)	Thinks can influence decisions affecting Britain	1686/7896 (21.35%)
	Thinks can influence decisions affecting local area	3027/7808 (38.77%)
Support for collective action (experience)	Participated in public meeting/rally/demonstration/protest/signed petition	759/8139 (9.33%)
*Social cohesion*		
Generalised trust (perceived)	Not worried about becoming a victim of crime	5226/8126 (64.31%)
	Trusts others in general	3501/8130 (43.06%)
Institutional trust (perceived)	Trusts the police	6744/8121 (83.04%)
	Trusts parliament	2710/8025 (33.77%)
	Trusts the local council	4869/7962 (61.15%)
Personal trust (perceived)	Trusts people in local neighbourhood	6606/7882 (83.81%)
Altruism (experienced)	Given help to group, club or organisation in the past 12 months	3309/8137 (40.67%)
	Helped someone who was not a relative in the past 12 months	4743/8137 (58.29%)
	Given money to charity in the past 4 weeks	6033/8129 (25.78%)
Horizontal networks (experienced)	Taken part in, supported or helped a club/group/organisation	4531/8138 (55.68%)
Cross-cutting ties (experienced)	Mixed socially with someone from different ethnic or religious background	6753/8137 (82.99%)
	Has friends from different ethnic groups	4582/8090 (56.64%)
	Has friends from different age groups	6031/8084 (74.60%)
National identity (perceived)	Feels a part of British society	7461/8083 (92.30%)
	Doesn’t think there is more racial prejudice than 5 years ago	3916/7600 (51.53%)
	Doesn’t think there is more religious prejudice than 5 years ago	3684/7346 (50.15%)
Community and local identity (perceived)	Agrees that people in the neighbourhood pull together to improve it	5017/7531 (66.62%)
	Feels they belong to the immediate neighbourhood	6151/8098 (75.96%)
	Thinks people in local area of different backgrounds get on well	5961/6977 (85.44%)
Interpersonal identity (perceived)	Family is very important to the sense of who they are	7127/8123 (87.84%)
*Socio*-*economic security*		
Income sufficiency (experienced)	Not cut back on food bills in last 12 months	6459/8115 (79.59%)
	Not cut back on utility bills in last 12 months	6332/8115 (78.03%)
Income security (experienced)	Not experienced a drop in income in last 12 months	5817/8115 (71.68%)
	Not fallen into arrears with bills or credit cards in last 12 months	7246/8115 (89.29%)
	Not fell into greater debt in last 12 months	7665/8115 (94.95%)
Income security (perceived)	Thinks financial circumstances will improve over next 12 months	2207/7852 (28.11%)
Housing security (experienced)	Not lost a home/fallen into arrears on rent/mortgage last 12 months	7776/8115 (95.82%)
	Not lost a home in last 12 months	8052/8115 (99.22%)
Employment security (experienced)	Not lost a job in the last 12 months	7599/8115 (93.64%)
Working conditions (experienced)	Worked 48 h a week or less if working full-time	3094/3817 (81.06%)
*Social inclusion*		
Constitutional/political rights (perceived)	Thinks they have all constitutional rights	3224/8107 (39.77%)
Civil rights (perceived)	Not worried about being attacked because of ethnicity or religion	7406/8114 (91.27%)
	Feels like treated with respect when using health services	7538/8108 (92.97%)
	Feels like treated with respect when using public transport	6378/7736 (82.45%)
Civil rights (experienced)	Not experienced harassment because of skin colour, ethnicity or religion in the last 2 years	7868/8136 (96.81%)
Labour market (experienced)	Has a paid job	5188/8139 (63.74%)

For Presentation, the table dichotomises indicators to show % of respondents with high social quality

It is important to note that the theory of social quality is not fully developed and issues remain with data adequacy, coherence, appropriateness and availability (van der Maesen and Walker 2012). Concretely, two of the main issues in applying the theory empirically are (1) existing data sources will inevitably only partly cover the breadth of the social quality framework and (2) there is some overlap of indicators across the four social quality conditional factors. Combined with the argument discussed earlier that a specific research agenda in area research appears to be the way forward, these empirical challenges suggest that the most logical way to research the relationship between social quality and health at least at this early stage is to analyse the sub-domains separately rather than create indexes or summary scores. This also allows future researchers to evaluate how we mapped the variables to the indicators and to replicate and build upon our findings.

### Analysis

We used random intercept multilevel ordered logit regression models to test for individual and neighbourhood social quality effects. Models were estimated using the generalized linear latent and mixed models (GLLAMM) procedure in Stata (Rabe-Hesketh et al. 2004). Random intercept instead of random coefficient models were specified because there was no strong theoretical reason to believe that the effects of social quality on health vary across different neighbourhoods. In addition, the main reason for using multilevel models was to test for contextual effects of social quality at the neighbourhood level. We therefore also specified cross-level interaction effects to test whether the effect of individual social quality on health depended on the level of social quality within the neighbourhood.

The Citizenship Survey sampled Output Areas as Primary Sampling Units (PSUs), selected these according to probability based on size, and then randomly selected addresses within PSUs. Output Areas contain an average of 123 households and can be taken as a proxy for neighbourhoods, as suggested by Poortinga (2012). The 8139 individuals in the sample were nested within 965 PSUs. The mean number of individuals per PSU was 8.4. Clarke and Wheaton (2007) use simulations to show that ‘unbiased and efficient estimates of fixed-effects and variance components can be obtained with 10 observations per group [even at low Intraluster Correlation Coefficient (ICC) values as long as there are at least 200 groups]’. (the ICC is a measure of how much variation in the dependent variable is explained by the clustering of groups.) Given there are a much larger number of PSUs in the sample, and that there are only 6 singleton neighbourhoods, bias and efficiency issues due to the structure of the sample are likely to be minimal.

## Results

The ICC of the null model showed that the differences between neighbourhoods accounted for 6.21% of the variance in self-rated health. As noted by Diez-Roux (2007), a low ICC value does not preclude important level 2 effects. With the social quality indicators as predictors, the ICC for the unadjusted models was around 6% and the neighbourhood variance was around .2 (full figures excluded from tables due to limited space, but available upon request). In the unadjusted analyses, with one exception (experienced openness/supportiveness of political system—discussed below), individual-level associations were nearly all in the direction consistent with social quality theory: higher levels of social quality were associated with higher levels of self-rated health. Only 3 of the 24 social quality domains tested failed to reach statistical significance. The strongest effect was for labour market experience, measured by whether the respondent had a paid job (coef. 1.08). For comparison, limiting long-term illness had a coefficient of 3.42 when regressed on self-rated health, meaning that not having a paid job has around a third of the negative effect on self-rated health compared with having a limiting long-term illness. Most of the significant coefficients were in the range of approximately .3–.6, or around 10% of the effect of having a limiting long-term illness. For nearly all indicators that were significant in the unadjusted analyses, controlling for socio-demographics and area-level factors generally attenuated their association with health to some extent, but they remained independently significant, suggesting that social quality indicators explain independent variation in self-rated health at the individual level.

In terms of neighbourhood-level effects, in the unadjusted models, 19/24 sub-domain effects on health were in expected direction, and 13/24 of the coefficients reached positive significance. These neighbourhood-level effects became insignificant once socio-demographics and area-level factors were added to the models, with one exception (experienced income sufficiency—outlined below). Cross-level interactions showed that in some cases, especially for indicators relating to experienced as opposed to perceived social quality, the neighbourhood level of social quality only had an effect for individuals who themselves had high levels of social quality, suggesting contextual effects. The neighbourhood-level effects are discussed in more detail below.

Focussing on the four separate conditional factors, firstly for social empowerment (Table 2) 3 of the 5 domains at the individual level were positively significantly associated with self-rated health (unadjusted analyses). Perceived availability of information, measured by literacy indicators, showed most association. Even after controlling for education and other socio-demographics, there was an independent positive effect at the individual level. Perceived application of knowledge, measured by a work skill indicator, showed a very similar albeit slightly weaker effect. Some neighbourhood effects for empowerment were present, evident in the openness/supportiveness of political system domains. For these variables, neighbourhood effects on health were stronger than individual effects. However in the final model the effects were attenuated, and there were negative individual effects for the experienced openness/supportiveness of political system domain. Lastly, the experienced support for collective action domain showed negligible effects, except a positive cross-level interaction. This suggests that taking part in collective action is only beneficial for health if others in your neighbourhood are doing the same. In sum, there is some evidence here that empowerment might be important for health at the individual level, and to a lesser extent at the neighbourhood level, especially with respect to the openness of the political system, and a supportive neighbourhood environment for collective action.

**Table 2:** Social empowerment

	Model 1 individ. SQ coef.	Model 2 individ. SQ coef.	Model 2 neighb. SQ coef.	Model 3 individ. SQ coef.	Model 3 neighb. SQ coef.	Model 4 individ. SQ coef.	Model 4 neighb. SQ coef.	Model 5 cross-level SQ coef.
Application of knowledge (perceived)	.216***	.210***	.017	.244***	.041	.238***	.025	−.210
Availability of information (perceived)	.447***	.454***	−.050	.312***	−.008	.331***	−.224	.108
Openness/supportiveness political system (experienced)	−.085**	−.126***	.398***	−.126**	.217	−.100**	.089	.321
Openness/supportiveness political system (perceived)	.385***	.337***	.394***	.288***	.213	.290***	.106	.091
Support for collective action (experienced)	.051	.016	.107**	−.087	.066	−.078	−.017	.337***

Model 1 IV/s: individual social quality

Model 2 IV/s: individual social quality, aggregate social quality

Model 3 IV/s: individual social quality, aggregate social quality, sex, age + age^2^, BME, income, qualification level, NS-SEC

Model 4 IV/s: individual social quality, aggregate social quality, sex, age + age^2^, BME, income, qualification level, NS-SEC, neighbourhood IMD, neighbourhood urban/rural status

** *p* < .05; *** *p* < .01

Social cohesion (Table 3) was more strongly associated with self-rated health than empowerment; at the individual level all coefficients were positive and significant (unadjusted analysis), and at the neighbourhood level 5/9 coefficients were positive and significant. The effects were strongest for the trust sub-domains especially, and to a lesser extent for the identity domains. There were also neighbourhood-level effects present for these domains, which were of around the same magnitude as the individual-level effects. Thus, in the unadjusted analyses, neighbourhood-levels of trust and identity have an independent association with self-rated health. One exception was for interpersonal identity, measured by a question asking about the importance of family for identity. Unexpectedly, there was a negative neighbourhood-level effect; the adjusted models suggest this may have been due to confounding of socio-demographic factors. For all models, adjusting for socio-demographics and area-level factors attenuated the individual-level effects somewhat, and completely attenuated the neighbourhood level effects. The positive effects of the indicators relating to experienced as opposed to perceived social quality—horizontal networks, altruism and cross-cutting ties were attenuated controlling for socio-demographics, suggesting the effects may have been due to compositional differences. Lastly, there were two cross-cutting effects here, suggesting that there was a particular health benefit for people who had high levels of personal trust (trust in neighbours) and cross-cutting ties (mixing with people from different backgrounds) in neighbourhoods with higher levels of these two indicators.

**Table 3:** Social cohesion

	Model 1 individ. SQ coef.	Model 2 individ. SQ coef.	Model 2 neighb. SQ coef.	Model 3 individ. SQ coef.	Model 3 neighb. SQ coef.	Model 4 individ. SQ coef.	Model 4 neighb. SQ coef.	Model 5 cross-level SQ coef.
Generalised trust (perceived)	.569***	.504***	.404***	.473***	.352***	.470***	.067	.219
Institutional trust (perceived)	.691***	.634***	.470***	.603***	.258	.594***	.058	−.017
Personal trust (perceived)	.380***	.317***	.288***	.467***	.288**	.440***	−.059	.522***
Altruism (experienced)	.268***	.237***	.182	.043	.261**	.049	−.108	.137
Horizontal networks (experienced)	.262***	.232***	.168	.089	.211	.096	−.056	−.216
Cross-cutting ties (experience)	.240***	.243***	−.018	−.124**	−.138	−.109**	−.075	.458**
National identity (perceived)	.436***	.396***	.313**	.329***	.156	.322***	.140	.185
Community and local identity (perceived)	.404***	.305***	.447***	.542***	.249	.526***	.039	.025
Interpersonal identity (perceived)	.228***	.262***	−.308**	.243***	−.203	.229***	−.221	−.004

Model 1 IV/s: individual social quality

Model 2 IV/s: individual social quality, aggregate social quality

Model 3 IV/s: individual social quality, aggregate social quality, sex, age + age^2^, BME, income, qualification level, NS-SEC

Model 4 IV/s: individual social quality, aggregate social quality, sex, age + age^2^, BME, income, qualification level, NS-SEC, IMD, neighbourhood IMD, neighbourhood urban/rural status

** *p* < .05; *** *p* < .01

For socio-economic security (Table 4), 4/6 domains were positively significantly associated with self-rated health at the individual level (unadjusted analyses). Housing security was most important for individual health. For all domains, once all controls were added to the models, significant individual-level effects remained. In terms of neighbourhood effects, these were overall less present for socio-economic security than the other domains. However, there were relatively strong effects for two domains—income insufficiency (cutting back on bills) and housing security (fallen into arrears or lost home). For housing insecurity, the coefficient becomes insignificant when controlling for area-level factors, but income sufficiency remains significant in the final model, suggesting that living in neighbourhoods with a higher level of income sufficiency is good for individual health. Furthermore, there were two significant cross-level interactions here, such that there was an additional health benefit for individuals with higher levels of income sufficiency and security if they lived in neighbourhoods with higher levels of these indicators. Two of the domains here had suppressor effects; that is, their association with health is only evident through their relationship with individual socio-demographic factors. As noted by Ludlow and Klein (2014), interpreting suppressor effects post hoc is of questionable utility. Nonetheless it does at least suggest these two domains are less clearly associated with health.

**Table 4:** Socio-economic security

	Model 1 individ. SQ coef.	Model 2 individ. SQ coef.	Model 2 neighb. SQ coef.	Model 3 individ. SQ coef.	Model 3 neighb. SQ coef.	Model 4 individ. SQ coef.	Model 4 neighb. SQ coef.	Model 5 cross-level coef.
Income sufficiency (experience)	.528***	.482***	.149***	.450***	.117**	.441***	.110**	.234**
Income security (experienced)	.400***	.381***	.207	.396***	.059	.388***	−.028	.562**
Income security (perceived)	.531***	.524***	.068	.309***	−.010	.297***	.044	−.002
Housing security (experience)	.737***	.618***	.204***	.600***	.162***	.591***	.095	.211
Employment security (experience)	.155	.174	−.035	.253***	−.018	.255***	−.032	.272
Working conditions (experienced)	.090	.173	−.382	.217**	−.408	.208**	−.318	−.038

Model 1 IV/s: individual social quality

Model 2 IV/s: individual social quality, aggregate social quality

Model 3 IV/s: individual social quality, aggregate social quality, sex, age + age^2^, BME, income, qualification level, NS-SEC

Model 4 IV/s: individual social quality, aggregate social quality, sex, age + age^2^, BME, income, qualification level, NS-SEC, IMD, neighbourhood IMD, neighbourhood urban/rural status

** *p* < .05; *** *p* < .01

Indicators of social inclusion (Table 5) were quite consistently associated with self-rated health; adjusting for socio-demographics and area-level factors had negligible effects on these associations. Civil rights, relating to discrimination, were slightly more associated with health than constitutional rights. Neighbourhood-level effects were present for both the constitutional/political rights domain and the labour market domain, though these were attenuated in the fully adjusted models. Labour market experience was particularly strongly associated with individual self-rated health (coef. 1.020 unadjusted analysis). This represents around a third of the effect on health as having a limiting long-term condition. There were no neighbourhood contextual effects here; individual experience of inclusion regardless of neighbourhood levels appears most important.

**Table 5:** Social inclusion

	Model 1 individ. SQ coef.	Model 2 individ. SQ coef.	Model 2 neighb. SQ coef.	Model 3 individ. SQ coef.	Model 3 neighb. SQ coef.	Model 4 individ. SQ coef.	Model 4 neighb. SQ coef.	Model 5 cross-level SQ coef.
Constitutional/political rights (perceived)	.491***	.439***	.374***	.387***	.225	.389***	.089	−.138
Civil rights (perceived)	.531***	.506***	.153	.593***	.167	.595***	.078	.219
Civil rights (experience)	.379***	.299**	.099	.567***	.065	.595***	−.004	.152
Labour market (experience)	1.076***	1.020***	.203***	.785***	.190***	.787***	.100	−.041

Model 1 IV/s: individual social quality

Model 2 IV/s: individual social quality, aggregate social quality

Model 3 IV/s: individual social quality, aggregate social quality, sex, age + age^2^, BME, income, qualification level, NS-SEC

Model 4 IV/s: individual social quality, aggregate social quality, sex, age + age^2^, BME, income, qualification level, NS-SEC, IMD, neighbourhood IMD, neighbourhood urban/rural status

** *p* < .05; *** *p* < .01

## Discussion

In this paper we tested the utility of social quality theory as a conceptual framework for examining individual- and neighbourhood-level influences on health. The underlying premise of this theory is that certain social conditions are necessary for social participation to enhance health and well-being. Social life involves interacting with a range of collectives, which requires that these collectivities—both formal and informal—are open (social inclusion). Through these interactions people experience social recognition from others, or a sense of social togetherness (social cohesion). They should also be enabled to play roles in and have a say in the development of society (social empowerment), and have security so that the process is not unduly precarious or risky (socio-economic security). Under social quality theory, these are marks of a high quality, health and well-being enhancing society.

It is clear that according to this theory there should exist some ‘out there’ level of social quality, hence our examination of area-level effects. We therefore discuss these results first, with the caveat that both the informal lifeworld and the formal world of systems are central to the theory; neighbourhood-level effects are one particular geographical focus and their examination cannot said to be representative of the breadth of the social quality framework.

We find that ultimately, individual-level experience of social quality is generally more important for individual-level health than neighbourhood-level social quality. We did find, however, that the neighbourhood context is important in how it interacts with individual social quality for the following indicators: experienced support for collective action (e.g. taking part in public meeting, rally, demonstration), perceived personal trust (neighbourhood trust), experienced cross-cutting ties (mixing with people from different backgrounds), income sufficiency (having enough to pay bills), and income security (not experienced a financial shock). The fact that the neighbourhood-level coefficients were not themselves significant suggests that higher levels of neighbourhood social quality provides an extra health benefit only to those individuals who themselves experience higher social quality, in the same way that Poortinga (2006) has suggested that social capital might function as collective resource people can draw on. Given that four out of the five significant cross-level interactions related to experience rather than perceptions of social quality, it is possible that this neighbourhood boost effect is only present for the former and not the latter, though the lack of data availability means this pattern should not be over-interpreted.

With respect to why neighbourhood-level effects were not found for the other social quality indicators, it might be that other spatial units are more pertinent, for example, at the regional or national level. In any case, the significant individual-level effects discussed below require social quality to be ‘out there’ in some capacity otherwise individual experiences of it would not be possible. For some indicators, it is logical that individual experience is much more important for health that neighbourhood-level (e.g. employment). In many cases, neighbourhood-level effects were attenuated controlling for IMD scores and urban/rural status. As noted by MacKinnon et al. (2000), it is impossible to differentiate whether this represents a mediation or confounding effect using cross-sectional data.

In terms of individual-level effects, the findings are generally consistent with previous research whilst also showing some novel results. Under social empowerment, it is well-established, for example, that indicators of knowledge and information (in this case, measured by job skills and literacy), are associated with health (though under social quality the causal mechanism is that these allow for individuals to have more power over their social lives). However, we also found that the perception that one is able to influence political life has an effect of roughly the same size. This issue does not seem to have been explored much empirically so far, yet may be especially pertinent given the wavering support for democracy following the financial crisis (Armingeon and Guthmann 2014).

The relationship between trust and health has received much attention in the literature. Thus we do not discuss our results in detail here, apart from re-iterating our finding centring on the neighbourhood contextual effect discussed above, and stating that our findings are consistent with previous research on both general trust (Subramanian et al. 2002) and institutional trust (Mohseni and Lindström 2008). The finding that neither altruism nor horizontal networks were associated with health once controlling for potential confounders especially at the area level suggests that some of the effect found in previous studies may be due to the influence of neighbourhood-level characteristics. Our finding that measures of identity were all associated with health is somewhat novel. It is useful here to distinguish civic from ethnic nationalism (Reeskens and Wright 2013), the former being inclusive and outward looking, consistent with the indicator analysed here. Reeskens and Wright found that this type of identity generates trust, which would help to explain the association we found. Community and local (in this case neighbourhood) identity is often conceptualised as bonding social capital, and has previously been shown to be important to health (Poortinga 2012). Although the health effects of interpersonal identity (in this case in relation to the family) do not appear to be examined empirically much so far, one possible causal mechanism is through its influence on work/life balance and satisfaction (Bagger et al. 2008).

Perhaps the conditional factor that has been previously researched the most is socio-economic security. We therefore discuss it less here except to say that the theoretical argument that security is necessary to social quality is supported by the consistent individual effects across all indicators. The most important indicator here was housing security, reflected by the size of the coefficient relative to the others. Similarly, income sufficiency is second in order of magnitude. These effects were significant even controlling for confounders, which is useful from a policy perspective since they represent more readily intervenable factors compared with social position. Socio-economic security is especially topical in relation to current debates on precarity—a state of uncertainty with an ever-present threat to livelihood—which has clear implications for both mental and physical health (Standing 2011).

The social inclusion domain also showed expected results with respect to unemployment; our finding here is consistent with the volume of previous research on this issue. The ‘rights’ aspect of social inclusion also showed strong effects, especially the civil rights indicators, which apart from unemployment had the strongest associations with self-rated health than any of the indicators in this analysis, which applied to both the experience and perception of these rights. The issue of rights in particular does not seem to have been examined much as a SDH before, despite its importance to public health equity being noted (Schrecker et al. 2010), and calls being made to establish suitable indicators (Gruskin and Ferguson 2009). Discrimination in particular, however, a key component of civil rights in the social quality framework, has previously been shown to be related to health, with the suggestion that depressive symptoms mediate the relationship (Todorova et al. 2010). A broader analysis of rights as a SDH seems to be warranted.

Overall this analysis suggests that various aspects of social quality are important for health. We have shown which indicators appear to be most important with respect to the neighbourhood context, and also which indicators are more important at the individual level. Our main findings can be summarised as follows:

- There are important neighbourhood contextual effects with respect to support for collective action, personal trust, cross-cutting ties, income sufficiency, and income security.
- At the individual level, the perception that one is able to influence political life has around the same strength of association with self-rated health as indicators of job skills and literacy.
- Measures of national, community and personal identity are aspects of social cohesion that are important for health alongside more researched indicators of trust.
- The security aspects of socioeconomic determinants are especially important: in order of magnitude, the strongest effects were for housing security, income sufficiency, and income security.
- Social rights, including institutional rights but especially civil rights, showed very large associations with self-rated health relative to the other indicators analysed here.

The overall policy focus of social quality is on factors that encourage, or make more difficult, the process of realising the social via full participation and social recognition. Social inequalities have long been held as fundamental causes of disease (Link and Phelan 1995), but a focus on socio-economic security in particular highlights factors amenable to intervention (e.g. household costs). Social quality moves beyond the socioeconomic sphere however to also show that issues of social empowerment, cohesion and inclusion can themselves be considered as fundamental causes. Fostering community and political participation, trust, and cross-cutting ties is likely to improve public health but crucially, only if it is considered how different people can access these collective resources. In addition, individuals’ rights, political efficacy and social identities present other promising novel avenues for social policy, and future research, to focus on.

Lastly, we note that further work on social quality and health is needed to: replicate findings; investigate the relationship between social position and social quality; focus on particular demographics (e.g. older people); incorporate ‘true’ level 2 variables (e.g. quality of local healthcare); investigate other levels of area effects beside the neighbourhood; use qualitative methods, and; consider other outcomes—for example, we would expect the underlying need for social recognition to have strong implications for mental health and well-being. However, social quality theory also needs further conceptual and empirical development [e.g. focussing on issues of data adequacy, coherence, appropriateness, and data availability (van der Maesen and Walker 2012)].

## Conclusion

Social quality theory lays out a broad framework for social epidemiological studies on SDH, underpinned by a sociological and social/policy-oriented theory that explicitly focuses on the nature of the social. It offers a potential answer to the critique of research in this area being empirically driven or theory-less. Since social quality is a theoretically-rooted framework centred on the social relations underpinning society, it offers an opportunity to move beyond individual socioeconomic status factors and consider holistic policy initiatives. In order to maintain the public health for all members of society, we should focus not only on the power and resources that flow from different positions in the social structure, but consider how society as a whole allows for and promotes empowerment, cohesion, inclusion, and socio-economic security.

## Limitations

Aggregating individual responses to at the neighbourhood level means that the analysis is potentially subject to same source bias (Diez-Roux 2007). Further work including true neighbourhood and other area-level factors such as levels of childcare, company policies and government budgets is necessary to investigate this further. Another limitation is that it is impossible to test for causality using cross-sectional data. More advanced causal designs are necessary to untangle the associations found in this analysis. Lastly it is important to keep in mind that data availability excluded those aged 70 + from the results which would have likely affected the findings. Because the purpose of this paper is to assess the utility of social quality theory in explaining health, a broad range of indicators are analysed, meaning there is not space to examine extremely detailed effects e.g. by examining non-linear or threshold effects, such as the heterogeneity of neighbourhoods or inequalities in social quality (see e.g. Galster 2012).

## Appendix

**Table:**

Domain/sub-domain	Indicators	Original variable name	Original range	Original missingness	Cleaned variable name	Cleaned range	Cleaned missingness
*Social empowerment*							
Application of knowledge (perceived)	Job uses skills/qualifications	SkiQual	3	2970 (2951 Item not applicable; 19 Don’t know)	skiqual_binary	2	2970
Availability of information (perceived)	Good at reading English	Reading	4	10 (Cannot read English)	Reading_REV	4	10
	Good at writing English	Writing	4	8 (Cannot write English)	Writing_REV	4	8
Summed variable	–	–	–	–	literacy	7	12
Openness/supportiveness political system (experience)	Taken part in a consultation about local services	PConsul4	2	2 Don’t know	PConsul4_BIN	2	2
	Contacted a political representative	PActUK	2	3 Don’t know	PActUK9_BIN	2	2
	Been a member of a group making decisions on services	zCivact2	2	0	zCivAct2	2	0
Summed variable	–	–	–	–	openpolsystemexp	4	3
Openness/supportiveness political system (perceived)	Thinks can influence decisions affecting Britain	PaffGB	4	243 Don’t know	PAffGB_REV	4	243
	Thinks can influence decisions affecting local area	PAffLoc	4	331 Don’t know	PAffLoc_REV	4	331
Summed variable	–	–	–	–	openpolsystemper	7	331
Support for collective action (experience)	Participated in public meeting/rally/demonstration/protest/signed petition	PRally4	2	1 Don’t know	PRally4_BIN	2	1
*Social cohesion*							
Generalised trust (perceived)	Not worried about becoming a victim of crime	WGenWorr	4	13 (Don’t know)	WGenWorr	4	13
	Trusts others in general	PTrust	3	9 (Don’t know)	Ptrust_REV	3	9
Summed variable	–	–	–	–	gentrust	6	22
Institutional trust (perceived)	Trusts the police	PTPolc	4	18 (Don’t know)	PTPolc_REV	4	18
	Trusts parliament	PTParl	4	114 (Don’t know)	PTParl_REV	4	114
	Trusts the local council	PTCncl	4	177 (Don’t know)	PTCncl_REV	4	177
Summed variable	–	–	–	–	institutionaltrust	10	268
Personal trust (perceived)	Trusts people in the local neighbourhood	STrust	4	257 (82 Don’t know; 175 Just moved here)	STrust_REV	4	257
Altruism (experience)	Given help to group, club or organisation in the past 12 months	FUnPd13	2	2 Don’t know	FUnPd13_REV	2	2
	Helped someone who was not a relative past 12 months	IHlp13	2	2 (1 Don’t know; 1 Refusal)	IHlp13_REV	2	2
	Given money to charity in the past 4 weeks	GGroup12a	2	10 (5 Don’t know; 5 Refusal)	GGroup12a_REV	2	14
Summed variable	–	–	–	–	altruism	4	13
Horizontal networks	Number of clubs, groups and organisations taken part in, supported or helped last 12 months	FGroup01-14	2	1 Refusal	horizontalnetworks	14	1
Cross-cutting ties (experience)	Mixed socially with someone from different ethnic or religious background	XMxoft	2	2 (Item not applicable)	XMxoft	2	8
	Has friends from different ethnic groups	SRace	4	49 (48 Don’t have any friends; 1 Refusal)	SRace_BIN	2	49
	Has friends from different age groups	SAge	4	55 (48 Don’t have any friends; 7 Don’t know)	SAge_BIN	2	55
Summed variable	–	–	–	–	crosscuttingties	4	57
National identity (perceived)	Feels a part of British society	FeBrit	4	56 Don’t know	FeBrit_REV	4	56
	Doesn’t think there is more racial prejudice today than 5 years ago	RPrej1	3	539 (538 Don’t know; 1 Refusal)	RPrej1_REV	3	539
	Doesn’t think there is more religious prejudice today than 5 years ago	Relinc	3	793 (791 Don’t know; 2 Refusal)	Relinc_REV	3	793
Summed variable	–	–	–	–	nationalidentity	8	1080
Community and local identity (perceived)	Agrees that people in the neighbourhood pull together to improve it	SPull	4	608 (544 Don’t know; 64 Nothing needs improving)	SPull_REV	4	608
	Feels they belong to the immediate neighbourhood	SBeNeigh	4	41 Don’t know	SBeNeigh_REV	4	41
	Thinks people in local area of different backgrounds get on well together	STogeth	4	1162 (1 Refusal; 307 Too few people in area; 449 All same backgrounds; 405 Don’t know)	STogeth_REV	4	1162
Summed variable	–	–	–	–	commandlocalidentity	10	1495
Interpersonal identity (perceived)	Family is important to the sense of who they are	ImpFam	4	16 (14 Don’t know; 2 Refusal)	ImpFam_REV	4	16
*Socio*-*economic security*							
Income sufficiency (experience)	Not cut back on food bills in last 12 months	FinHap_9	2	24 (9 Don’t know; 15 Refusal)	FinHap_9_BIN	2	24
	Not cut back on utility bills in last 12 months	FinHap_10	2	24 (9 Don’t know; 15 Refusal; 9 Item not applicable)	FinHap_10_BIN	2	24
Summed variable	–	–	–	–	incomesuff	2	24
Income security (experienced)	Not experienced a drop in income in last 12 months	FinHap_1	2	24 (9 Don’t know; 15 Refusal; 9 Item not applicable)	FinHap_1_BIN	2	24
	Not fallen into arrears with bills or credit cards in last 12 months	FinHap_2	2	24 (9 Don’t know; 15 Refusal; 9 Item not applicable)	FinHap_2_BIN	2	24
	Not fall into greater debt in last 12 months	FinHap_6	2	24 (9 Don’t know; 15 Refusal; 9 Item not applicable)	FinHap_6_BIN	2	24
Summed variable	–	–	–	–	incomesecexp	4	24
Income security (perceived)	Thinks financial circumstances will improve over next 12 months	FinCirc	5	287 (284 Don’t know; 3 Refusal)	FinCirc_REV	5	287
Housing security (experience)	Not fallen into arrears on rent/mortgage in last 12 months	FinHap_3	2	24 (9 Don’t know; 15 Refusal)	FinHap_3_BIN	2	24
	Not lost a home in last 12 months	FinHap_7	2	24 (9 Don’t know; 15 Refusal)	FinHap_7_BIN	2	24
Summed variable	–	–	–	–	housingsecurity	2	24
Employment security (experience)	Not lost a job in the last 12 months	FinHap_8		24 (9 Don’t know; 15 Refusal)	FinHap_8_BIN	2	24
Working conditions (experienced)	Number of hours worked per week for current full-time workers	Usuhr2	81	2958 (Not applicable)	Usuhr2_CLEAN	81	2958
*Social inclusion*							
Constitutional/political rights (perceived)	Number of constitutional rights respondent believes they have	EHave01-09	2	32 (30 Don’t know; 2 Refusal)	EHave_REV	10	32
Civil rights (perceived)	Not worried about being attacked because of skin colour, ethnicity or religion	WRaceAtt	4	25 Don’t know	WRaceAtt	4	25
	Feels like treated with respect when using health services	ReHeal	5	31 (30 Don’t know; 1 Refusal)	ReHeal_REV	5	31
	Feels like treated with respect when using public transport	RePub	5	403 (400 Don’t know; 3 Refusal; 7 Item not applicable)	RePub_REV	5	403
Summed variable					civilrightsper	11	452
Civil rights (experience)	Not experienced harassment because of skin colour, ethnicity or religion in the last 2 years	SHrsmnt	2	3 (2 Don’t know; 1 Refusal)	SHrsmnt	2	3
Labour market (experience)	Has a paid job	DWorkA	2	0	DWorkA_BIN	2	0
